# Association and biomarker potential of elevated serum adiponectin with nephropathy among type 1 and type 2 diabetics: A meta-analysis

**DOI:** 10.1371/journal.pone.0208905

**Published:** 2018-12-17

**Authors:** Noel Pabalan, Raphael Enrique Tiongco, Jefferyl Kae Pandac, Noemi Anne Paragas, Shamar Lo Lasta, Nelven Gallego, Hamdi Jarjanazi, Maria Ruth Pineda-Cortel

**Affiliations:** 1 Center for Research and Development, Angeles University Foundation, Angeles City, Philippines; 2 Department of Medical Technology, College of Allied Medical Professions, Angeles University Foundation, Angeles City, Philippines; 3 Graduate School, University of Santo Tomas, Manila City, Philippines; 4 Institute of Clinical Laboratory Sciences, Silliman University, Dumaguete, Philippines; 5 Department of Medical Technology, Faculty of Pharmacy, University of Santo Tomas, Manila City, Philippines; 6 Environmental Monitoring and Reporting Branch, Ontario Ministry of the Environment and Climate Change, Toronto, Ontario, Canada; 7 Research Center for the Natural and Applied Sciences, University of Santo Tomas, Manila City, Philippines; Universita degli Studi di Perugia, ITALY

## Abstract

**Background:**

Managing nephropathy associated with diabetes mellitus warrant investigation of relevant biomarkers in predicting this condition. Adiponectin (ADP) may hold promise as a biomarker for diabetic nephropathy (DN). In this study, we examine associations of ADP with DN by meta-analyzing relevant literature. We also examined the predictive potential of ADP and estimate progression of DN.

**Methods:**

Multi-database literature searches and serial omissions of articles yielded 13 studies for inclusion in the meta-analysis. We compared ADP levels between controls/ normoalbuminuria and cases with micro- and macroalbuminuria (MI and MA, respectively) as well as MI versus MA using standardized mean differences (SMD). Associations of ADP with DN were indicated with the P-value considered significant at ≤ 0.05. Subgrouping was based on diabetes type (1 and 2). Predictive potential of ADP was explored with AUC (area under the curve) derived from Receiver Operating Characteristic curve analysis.

**Results and conclusion:**

At high P-values of <10^−5^, overall and subgroup outcomes indicated ADP associations with DN (up to SMD = 1.89–2.26, respectively). However, heterogeneity of the initial SMD effects (up to I^2^ = 99%) warranted examination of their sources which with the Galbraith plot method, either eliminated or reduced their heterogeneity, signifying combinability of the studies. This feature along with consistency of significant associations, robust outcomes and significant AUC values provide good evidence of the associative and predictive roles of ADP in DN.

## Introduction

Diabetes mellitus (DM) is a global health problem affecting an estimated 422 million people. This estimate may increase to about 48% by the year 2045 [1]. A salient feature of DM is the increasing prevalence among children, adolescents and younger adults warranting early and effective screening of this disease [2]. A serious microvascular complication of DM is diabetic nephropathy (DN), which occurs in 30–50% of patients with type 1 (T1) and type 2 (T2) DM. A widely used screening tool for DN is measurement of urine albumin in a 24-hour sample. A level of urine albumin at > 30mg/day suggests DN [3]. Molecular mechanisms for DN are currently unclear, but adiponectin (ADP) may play a role in DN pathogenesis [4]. ADP is a small collagen-like protein encoded by the adiponectin gene, which is primarily expressed by adipocytes [5]. ADP is critical in glucose and fat metabolism where it regulates glucose uptake, gluconeogenesis and fatty acid oxidation in hepatocytes [6]. ADP has been shown to have anti-inflammatory, insulin-sensitizing, anti-atherogenic and cardioprotective activities [7–9] and has been linked to insulin resistance, obesity and cardiovascular diseases [10]. Patients at risk for these complications were found to have lower serum ADP levels [11]. In contrast, elevated ADP levels were found to be an independent predictor of the progression to end-stage renal disease (ESRD) [12]. Glomerular damage may be facilitated with passage of ADP through the glomerular basement membrane and excreted in the urine. Indeed, various ADP isoforms (low, medium and high molecular weights) can be measured in the urine, where ADP is considered to be a marker of vascular damage [13, 14]. Reports of ADP effects on DN have been variable, which prompted a meta-analysis to obtain more precise estimates. Here, we provide meta-analytical evidence to associate ADP with DN as well as examine biomarker potential of ADP.

## Materials and methods

### Search strategy

Literature search and article selection using the terms, “adiponectin”, “albuminuria” and “diabetic nephropathy” in English, we searched MEDLINE using PubMed, Science Direct and Google Scholar for publications as of March 07, 2018 (S1 Table). References cited in the retrieved publications were screened manually to identify additional eligible articles. Inclusion criteria included articles that presented ADP data with Mogensen’s design for albuminuria and DN [15].

### Data extraction and eligibility criteria

Two investigators (NP and HJ) independently extracted data and reached consensus on all the items. For each eligible study, the following information was extracted: the first author's name, publication year, country, ethnicity, patient sample size, source of samples, type of diabetes and whether the article was included in a previous meta-analysis [16]. Studies eligible for meta-analysis would have reported data for mean ADP (serum or plasma) concentrations both in patients with albuminuria (case) and without (controls, [CN]) or its levels below 30 mg/day (normoalbuminuria, [NO]). Studies were excluded from our meta-analysis if they did not specifically report ADP concentrations in patients in terms of Mogensen’s criteria [15]. In addition, we focused on studies with patients targeted for secondary prevention. Study authors were contacted to obtain additional information.

### Methodological quality of the studies

The Newcastle–Ottawa Scale (NOS) assessment tool [17] was used to evaluate methodological quality of the included studies. These studies were judged based on three broad perspectives: selection, comparability and exposure (S2 Table). The star rating system has scores ranging from zero (worst) to 9 (best). Scores of 5–6 and ≥7 stars indicate moderate and high quality, respectively.

### Meta-analysis protocol

The relationship between ADP and DN was expressed as standardized mean difference (SMD) and 95% confidence intervals (CI) using Review Manager 5.3 (Copenhagen: Nordic Cochrane Centre, Cochrane Collaboration, 2014). SMD estimates were considered as statistically significant if their CI did not cross zero and their P-values were < 0.05. Most of the papers presented median and range, which were converted into approximate mean and standard deviation (SD) following Hozo et al. [18]. Where mean and 95% CI or standard error were reported, SD were derived as described by the Cochrane Collaboration [19]. From albuminuria data in DN patients, we compared NO with microalbuminuria (MI); NO with macroalbuminuria (MA) and MI with MA. Where CN were explicitly present, we also compared these with MI and with MA, respectively. Pooled estimates were obtained using either the fixed [20] (absence of heterogeneity) or random [21] (in its presence) effects models. We used random-effects model when results of the χ2-based Q test [22] yielded P-values of < 0.10 [23]. Sources of heterogeneity were identified with outlier analysis using the Galbraith plot method [24]. Heterogeneity was quantified with the I^2^ statistic which measures the degree of inconsistency among studies [25]. Pooled estimates were subjected to sensitivity analysis which involved omitting one study at a time followed by recalculation to test for robustness of the summary effects. In subgroup analysis, we were confronted with ethnicity and diabetes type [DT], both with equal logistic potential for analysis (number of studies). We opted for DT (1 and 2) because it was less heterogeneous than ethnicity, which was beset with different geographies and admixture. Publication bias assessment focused only on comparisons with the number of studies > 10 because lower than this number, sensitivity of the qualitative and quantitative tests are low [26]. With SigmaPlot 11.0 (Systat Software, San Jose, CA), we used the ROC (Receiver Operating Characteristic) curve to estimate potential of ADP in predicting DN. This curve yields AUC (area under the curve) values (range: zero to one) that indicate degree of predictive accuracy and P-values to indicate presence or absence of significance. All P-values were two-sided with significance set at ≤ 0.05 except in heterogeneity estimation.

## Results

### Search outcomes

**Fig 1:**
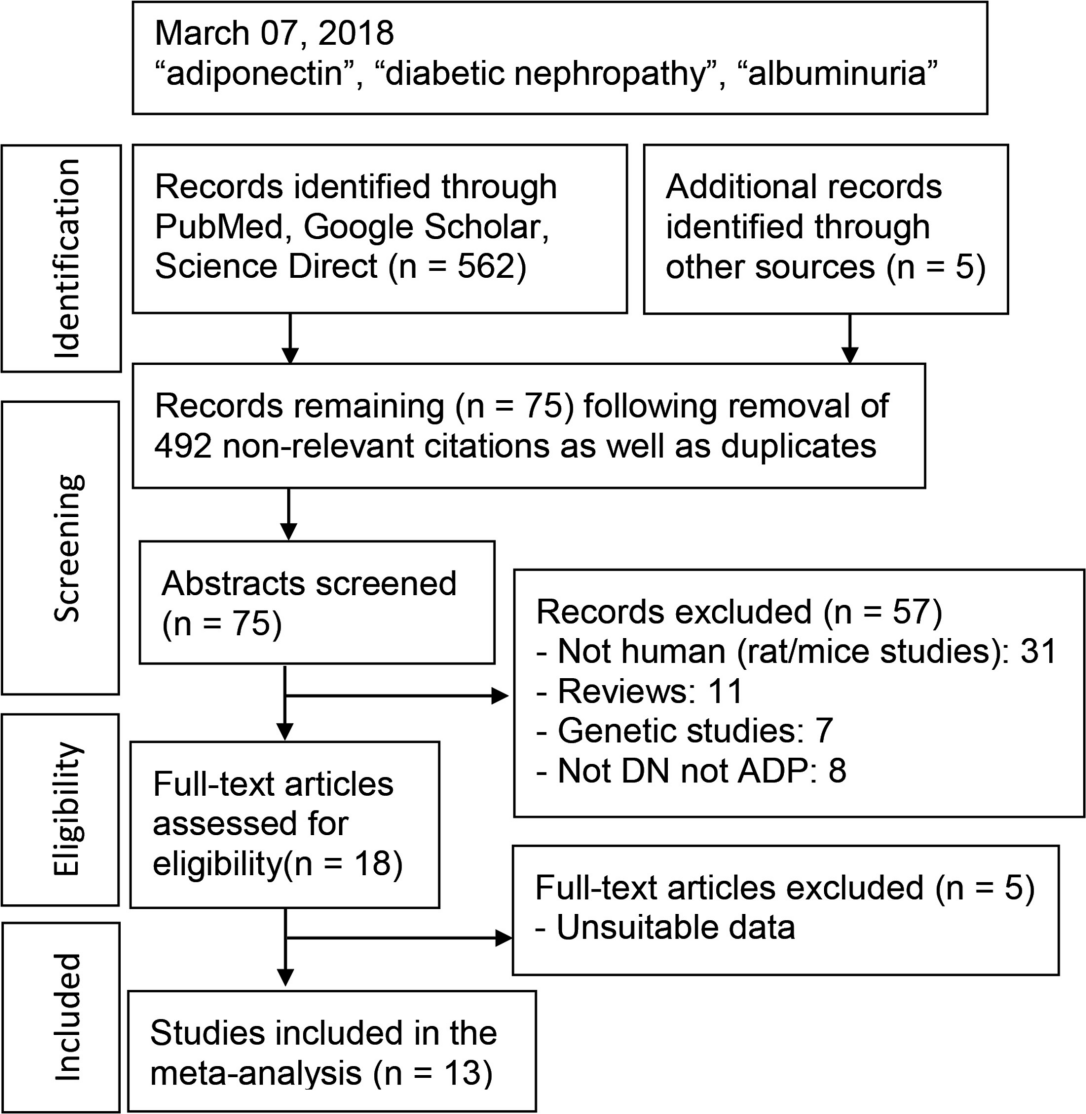
Summary of literature search: DN: diabetic nephropathy; ADP: adiponectin.

Fig 1 outlines the study selection process in a flowchart following PRISMA (Preferred Reporting Items for Systematic Reviews and Meta-Analyses) guidelines [27]. A total of 567 citations during the initial search were followed by a series of omissions (S1 List) that eventually yielded 13 studies for inclusion in the meta-analysis [28–40].

### Characteristics of the studies

Table 1 features characteristics of the included publications, the years of which ranged from 2004 to 2013. Overall, the total sample size included in the meta-analysis was 5,632 with a wide range among patients across all studies (38 to 2,090). In terms of DT, seven examined T2DM [28–33, 40] with remaining six focused on T1DM [34–39]. This meta-analysis included eight studies [29, 33–39] not found in the Rodriguez et al. meta-analysis [16]. S3 Table summarizes additional qualitative features of the component articles that include clinical and study design parameters. In clinical terms, patient diagnoses and restrictions to the cases were compared between studies. NOS scoring showed the mean and SD to be 6.2 ± 1.2 and a median of 6 indicating that the included studies were of moderate quality. S2 Table details the scoring outcomes from each included study. The PRISMA checklist was generated to provide detailed description of this meta-analysis (S4 Table).

**Table 1:** Characteristics of the included studies.

First author	[R]	Year	Country	Ethnicity	Sample size	Source	DT	Comparisons (n)	AJR	NOS
Ran	[28]	2010	China	Asian	50	Plasma	2	CN/NO* (18) MI (17) MA (15)	Yes	7
Fujita	[29]	2006	Japan	Asian	53	Serum	2	CN (20) NO (19) MI (18)	Yes	6
Kato	[30]	2008	Japan	Asian	198	Serum	2	NO (116) MI (47) MA (24)	Yes	4
Komaba	[31]	2006	Japan	Asian	179	Serum	2	NO (86) MI (44) MA (23)	Yes	5
Koshimura	[32]	2004	Japan	Asian	38	Serum	2	NO (18) MI (7) MA (13)	Yes	7
Saito	[33]	2007	Japan	Asian	280	Serum	2	CN (49) MA (22)	Yes	6
Jorsal	[34]	2013	Denmark	Western	145	Serum	1	CN (55) NO (58) MI (43) MA (44)	No	5
Schalkwijk	[35]	2006	Europe	Western	543	Serum	1	NO (328) MI (82) MA (128)	No	5
Panduru	[36]	2015	Finland	Western	2,090	Serum	1	CN (111) NO (1451) MI 319) MA (320)	No	7
Saraheimo	[37]	2008	Finland	Western	1,330	Serum	1	CN (204) NO (818) MI (216) MA (296)	No	6
Saraheimo	[38]	2005	Finland	Western	189	Serum	1	NO (66) MI (63) MA (60)	No	7
Hadjadj	[39]	2005	France	Western	126	Plasma	1	MI (18) MA (108)	No	8
Yilmaz	[40]	2008	Turkey	Western	411	Serum	2	CN (38) MI (40) MA (45)	Yes	8

[R] Reference number; DT: diabetes type; n: number of studies (n = 13); CN: control; NO: normoalbuminuria; MI: microalbuminuria; MA: macroalbuminuria; CN/NO*: NO is CN; AJR: Rodriguez et al. meta-analysis (yes and no indicate that the article was either in AJR or not, respectively); NOS: Newcastle-Ottawa Scale

## Meta-analysis findings

### Overall analysis

Table 2 summarizes the initial overall findings which indicate significantly higher ADP levels in MI and MA when compared to CN or NO (SMD = 0.74–1.89, P < 10^−5^–0.03). However, these pre-outlier outcomes were all highly heterogeneous (P_b_ < 10^−5^, I^2^ = 94–99%) with values obtained under the random-effects model. This heterogeneity warranted examining their sources, so we applied the outlier treatment which impacted on significance (reduced or elevated) and heterogeneity (eliminated or diminished) of the findings. The post-outlier values were thus, predominantly fixed-effects.

**Table 2:** Overall summary associations of adiponectin with diabetic nephropathy.

		Test of association			Test of heterogeneity					Test of association			Test of heterogeneity			
	n	SMD	95% CI	P_a_	P_b_	I^2^ (%)	AM	NSO (%)	n	SMD	95% CI	P_a_	P_b_	I^2^ (%)	AM	Effects of outlier treatment
**Pre-outlier**									**Post-outlier**							
CN v MI	6	0.73	-0.29, 1.74	0.16	10^−5^	98	R	3 (50)	3	**1.19**	**0.41, 1.97**	**0.003**	10^−5^	96	R	NCH, GS
CN v MA	7	**0.74**	**0.07, 1.41**	**0.03**	10^−5^	97	R	3 (43)	4	**0.88**	**0.75, 1.02**	**10**^**−5**^	0.76	0	F	EH, MHS
NO v MI	10	**0.82**	**0.21, 1.43**	**0.009**	10^−5^	98	R	4 (40)	6	**0.15**	**0.03, 0.27**	**0.01**	0.22	28	F	RH, HMS
NO v MA	10	**1.89**	**1.04, 2.73**	**10**^**−5**^	10^−5^	99	R	3 (30)	7	**0.82**	**0.70, 0.93**	**10**^**−5**^	0.14	38	F	RH, RHS
MI v MA	12	**0.68**	**0.24, 1.13**	**0.003**	10^−5^	94	R	6 (50)	6	**0.55**	**0.42, 0.69**	**10**^**−5**^	0.44	0	F	EH, MHS

CN: control; NO: normoalbuminuria; MI: microalbuminuria; MA: macroalbuminuria; v: versus; n: number of studies in the comparison; SMD: standardized mean difference; CI: confidence interval; P_a_: P-value for association; P_b_: P-value for heterogeneity; AM: analysis model; R: random-effects; F: fixed-effects; NSO: number of studies omitted; NCH: no change in heterogeneity; EH: eliminated heterogeneity; RH: reduced heterogeneity; GS: gain in significance; MHS: moderate to high significance; HMS: high to moderate significance; RHS: retained high significance

**Fig 2:**
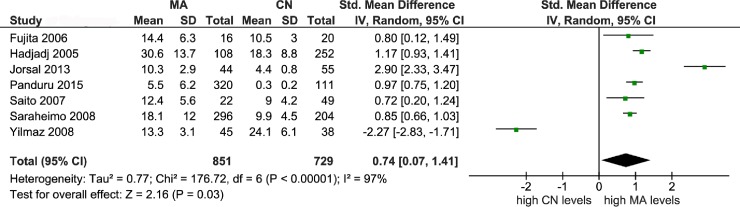
Forest plot of control versus macroalbuminuria comparison. LEGEND: MA: macroalbuminuria; CN: control; SD: standard deviation; CI: confidence interval; df: degree of freedom.

**Fig 3:**
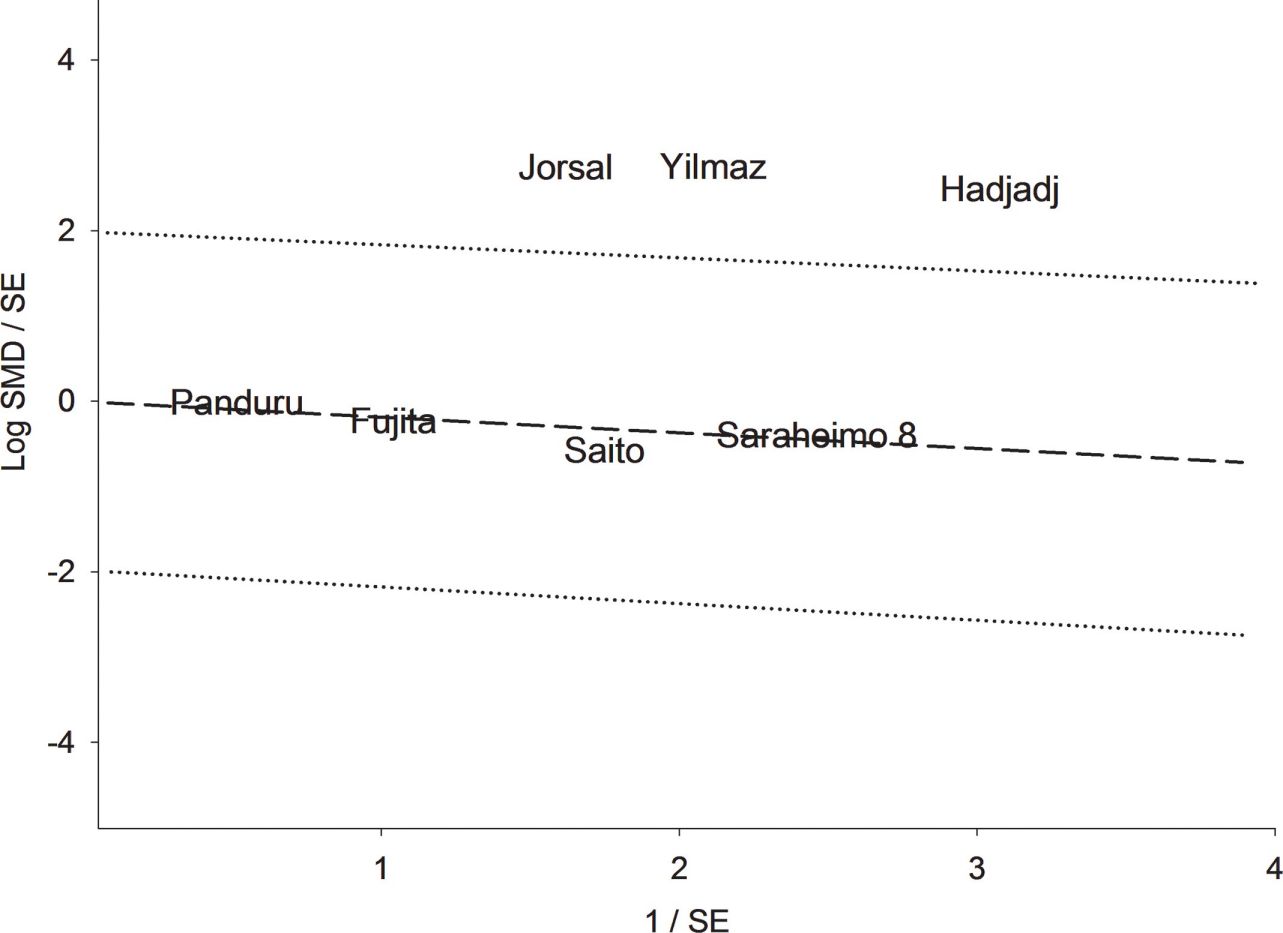
Galbraith plot analysis of outliers from the control versus macroalbuminuria comparison. LEGEND: SMD: standardized mean difference; SE: standard error.

**Fig 4:**
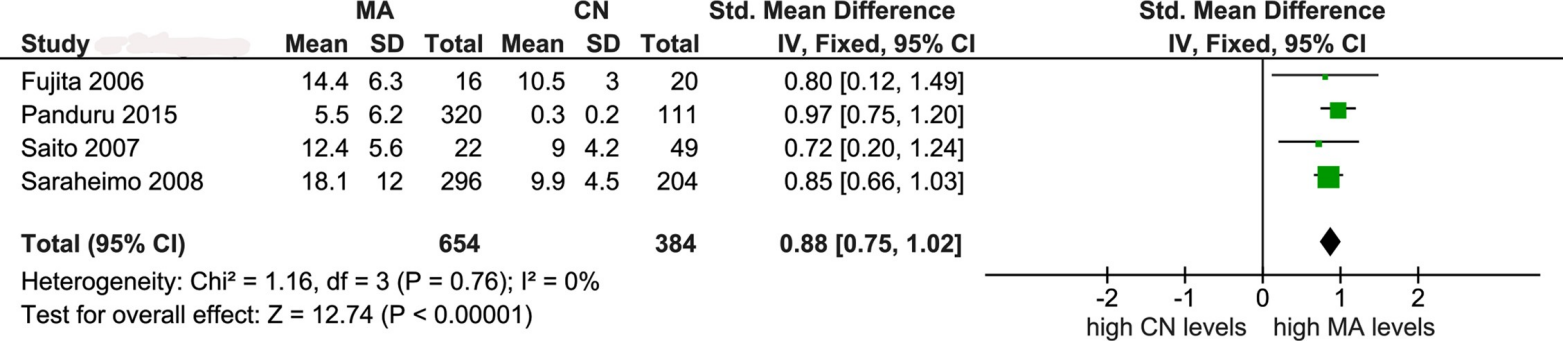
Forest plot of post-outlier control versus macroalbuminuria comparison. LEGEND: MA: macroalbuminuria; CN: control; SD: standard deviation; CI: confidence interval; df: degree of freedom.

Figs 2–4 illustrate the mechanism of outlier treatment. Fig 2 details how the overall pooled SMD was derived from the CN versus MA comparison. In this forest plot, the random pooled effect was modestly significant (SMD = 0.74, P_a_ = 0.03) and highly heterogeneous (P_b_ < 10^−5^, I^2^ = 97%). Sources of this heterogeneity were examined with the Galbraith plot (Fig 3) which identified three outlier studies [34, 39, 40]. Outlier treatment outcomes were (i) eliminated heterogeneity (P_b_ = 0.76, I^2^ = 0%), (ii) high statistical significance (SMD = 0.88, P_a_ < 10^−5^) and (iii) shift to fixed-effects model (Fig 4).

### Subgroup analysis

Table 3 shows all pre-outlier outcomes in the DT subgroup. Significant T1DM subgroup effects indicated higher ADP levels in MI and MA compared to either CN or NO (SMD = 0.75–2.26, P_a_ = 10^−5^–0.03). T2DM SMD outcomes ranged from -1.44 to 1.40 (P_a_ < 10^−5^) indicating variability in the direction of effects. This variability disappeared following outlier treatment (SMD = 0.56–1.15, P_a_ = 10^−5^–0.001). Where subgroup effects were heterogeneous, their sources were examined with outlier treatment, outcomes of which are summarized in Table 4. All post-outlier outcomes in the subgroups were homogeneous (I^2^ = 0%) regardless of magnitude of associative significance (P_a_ < 10^−5^–0.006).

**Table 3:** Subgroup analysis of adiponectin associations with diabetic nephropathy.

		Test of association			Test of heterogeneity		
	n	SMD	95% CI	P_a_	P_b_	I^2^ (%)	AM
**T1DM**							
CN v MI	4	**1.74**	**0.78, 2.69**	**0.0004**	10^−5^	97	R
CN v MA	4	**1.39**	**0.88, 1.89**	**10**^**−5**^	10^−5^	93	R
NO v MI	5	**1.10**	**0.13, 2.07**	**0.03**	10^−5^	99	R
NO v MA	5	**2.26**	**0.97, 3.56**	**0.0006**	10^−5^	99	R
MI v MA	6	**0.75**	**0.22,1.29**	**0.006**	10^−5^	95	R
**T2DM**							
CN v MI	2	**-1.44**	**-1.85, -1.03**	**10**^**−5**^	0.48	0	F
CN v MA	3	-0.25	-2.28, 1.78	0.81	10^−5^	97	R
NO v MI	5	**0.46**	**0.25, 0.68**	**10**^**−5**^	0.43	0	F
NO v MA	5	**1.40**	**0.93, 1.87**	**10**^**−5**^	0.03	62	R
MI v MA	6	0.64	-0.29, 1.57	0.18	10^−5^	92	R

T1DM: Type 1 diabetes mellitus; T2DM: Type 2 diabetes mellitus; CN: control; NO: normoalbuminuria; MI: microalbuminuria; MA: macroalbuminuria; v: versus; n: number of studies; SMD: standardized mean difference; CI: confidence interval; P_a_: P-value for association; P_b_: P-value for heterogeneity; AM: analysis model; F: fixed-effects, R: random-effects

**Table 4:** Outlier outcomes in the subgroups.

		Test of association			Test of heterogeneity		
	n	SMD	95% CI	P_a_	P_b_	I^2^ (%)	AM
**T1 DM**							
CN v MA	2	**0.90**	**0.75, 1.04**	**10**^**−5**^	0.40	0	F
NO v MI	2	**0.37**	**0.11, 0.63**	**0.006**	0.47	0	F
NO v MA	3	**0.76**	**0.63, 0.88**	**10**^**−5**^	0.51	0	F
MI v MA	2	**0.61**	**0.45, 0.77**	**10**^**−5**^	0.46	0	F
**T2 DM**							
CN v MA	2	**0.75**	**0.34, 1.16**	**0.0004**	0.85	0	F
NO v MA	4	**1.15**	**0.86, 1.43**	**10**^**−5**^	0.54	0	F
MI v MA	3	**0.56**	**0.23, 0.90**	**0.001**	0.85	0	F

T1DM: Type 1 diabetes mellitus; T2DM: Type 2 diabetes mellitus; CN: control; NO: normoalbuminuria; MI: microalbuminuria; MA: macroalbuminuria; v: versus; n: number of studies; SMD: standardized mean difference; CI: confidence interval; P_a_: P-value for association; P_b_: P-value for heterogeneity; AM: analysis model; F: fixed-effects; CN versus MI in T1DM did not change with outlier treatment.

Table 5 presents the trends from pre- to post-outlier outcomes using three indicators. In the overall analysis, proportion of high significance (P ≤ 10^−5^) increased (20% to 60%), heterogeneity (random-effects) declined (100% to 20%) and homogeneity (I^2^ = 0%) was generated (0% to 40%). In the subgroup comparisons, high significance and homogeneity frequencies increased (40% to 57% and 20% to 100%, respectively). Most salient here was disappearance of heterogeneity (80% to 0%).

**Table 5:** Summary of outlier outcomes by indicator.

			Overall ^a^			Subgroup		
Indicator	Feature	n	Pre (%)	Post (%)	n	Pre ^b^ (%)	n	Post ^c^ (%)
P ≤ 10^−5^	high significance	5	1 (20)	3 (60)	10	4 (40)	7	4 (57)
Random-effects	heterogeneity	5	5 (100)	1 (20)	10	8 (80)	7	0 (0)
I^2^ = 0%	homogeneity	5	0 (0)	2 (40)	10	2 (20)	7	7 (100)

n: number of indicators

^a^: data from Table 2

^b^: data from Table 3

^c^: data from Table 4; Pre; pre-outlier; Post: post-outlier

### Sensitivity analysis and publication bias

Outcomes from all levels of comparisons were found to be robust indicating the stability of our findings (data not shown). Table 6 shows that all comparisons with studies >10 showed no evidence of publication bias, except the NO versus MA comparison in the correlation test (P < 0.001).

**Table 6:** Publication bias assessment of adiponectin association outcomes with diabetic nephropathy.

		Egger regression asymmetry test		Begg-Mazumdar correlation test	
	n	Intercept	P-value	Kendall'sτ	P-value
NO v MI	10	1.69	0.68	0.20	0.42
NO v MA	10	3.35	0.49	0.49	**< 0.001**
MI v MA	12	-1.07	0.68	-0.03	0.89

NO: normoalbuminuria; MI: microalbuminuria; MA: macroalbuminuria; v: versus; n: number of studies

### Evaluating the potential of ADP in predicting DN

**Fig 5:**
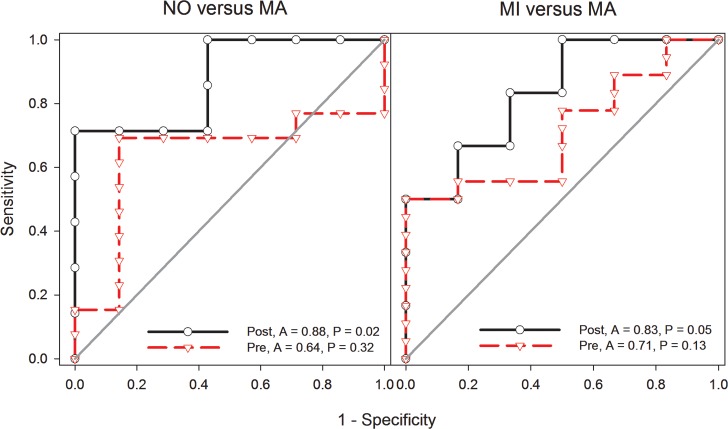
Receiver Operating Characteristic (ROC) curve analysis of ADP potential to predict DN. Area under the curve (A) values (range: zero-one) indicate predictive potential classified as poor (< 0.69), fair (> 0.70–0.79) and good (0.80–0.89). P-values associated with AUC are significant at ≤ 0.05. ADP: adiponectin; DN: diabetic nephropathy; NO: normoalbuminuria; MA: macroalbuminuria; A: area under the curve; Pre: pre-outlier; Post: post-outlier.

We generated ROC curves for the CN, NO, MI and MA comparisons, details of which are summarized in S5 Table. Those with significant AUC outcomes are shown in Fig 5. ROC curve for ADP (NO versus MA and MI versus MA) showed respective post-outlier (non-heterogeneous) AUC values of 0.88 (95% CI 0.68–1.07, P = 0.02) and 0.83 (95% CI 0.59–1.10, P = 0.05) indicating good potential in discriminating patients with and without DN.

## Discussion

### Summary interpretations

The meta-analysis findings in this study provide good evidence of associative and predictive potential of ADP, for two reasons. First, consistent outcomes of significance were supported by homogeneity of post-outlier results indicating combinability of the studies. Furthermore, high magnitude (multiple P_a_-values of 10^−5^), precision of effects (indicated by the narrow CIs), robustness (from sensitivity treatment) and lack of bias (outcomes of Egger’ tests) helped improve the outcomes. Key indicators used in this meta-analysis rendered outlier treatment as instrumental in generating high significance and homogeneity as well as eliminating heterogeneity. In addition, outlier treatment generated associative similarities between T1DM and T2DM by virtue of common high significance (P ≤ 10^−5^) and homogeneity (I^2^ = 0%). Second, the difference in AUC (NO versus MA and MI versus MA) between the non-significant pre-outlier outcomes (P = 0.32 and 0.13) and significant post-outlier results (P = 0.02 and 0.05) indicates the contributive utility of outlier treatment to the discriminative potential of ADP in predicting DN (NO versus MI) and estimating its progression (MI versus MA). However, marginal significance of the MI versus MA outcome warrant caution in its interpretation.

Primary study findings on the role of ADP in DN have been variable. Several methodological problems may explain the discrepancies, including limited statistical power, unrecognized confounding factors and stratification of populations [41]. Reporting study-specific effects of ADP has ranged from association to prediction and progression. In presence of association, risk effects may indicate protection or susceptibility which may or may not be significant. Meta-analysis, however, informs more in reporting effects for the role of ADP in DN. These involve exploration of magnitude, precision, consistency, stability and heterogeneity of outcomes. These features, along with significant AUC outcomes, raise the levels of evidence to support conclusions of ADP-DN associations and potential of ADP in helping predict DN progression.

### Comparison with previous studies

Significant AUC outcomes in our post-outlier analysis likely render a role for ADP as susceptibility screening tool for DN. Of the five component articles that addressed the role of ADP in DN progression or its predictive capability [28, 29, 36, 37, 40], one used ROC curve analysis but focused on uADP [36]. An external study [42] that examined AUC in predicting renal decline in pediatric lupus nephritis also used uADP. In terms of direction of effect and significance of association, our DN findings agree with a previous meta-analysis [16]. Still, differences exist between the two meta-analyses (previous and ours). Rodriguez et al. [16] covered four studies examining ADP and DN in three milieus (NO, MI and MA) without the actual CN data. We covered 13 studies, using CN where data were available. In terms of levels of comparisons, the previous meta-analysis covered three (NO versus MI; NO versus MA and MI versus MA) while we covered up to five which included CN. Of the five levels of comparisons in the overall analysis and subgroups, NO versus MA had the strongest associations as indicated by SMDs of up 2.3-fold and the most number of 10^−5^ P_a_-value (n = 7). This finding agrees with that of Rodriguez et al. [16] (SMD = 1.37, P < 10^−5^). In contrast to the previous meta-analysis, however, our reason for exclusive focus on ADP was so that we could apply a number of meta-analytical treatments to our findings, endeavoring to raise the quality of evidence. The findings from both meta-analyses (theirs and ours) were not materially different (both were statistically significant). What differentiates our findings is that we explore the heterogeneous outcomes with outlier analysis, thus generating pooled SMDs of either non-heterogeneous or homogeneous nature. Thus, we address combinability of the studies in addition to consistency (significance across comparisons), stability (subgroup analysis) and robustness (sensitivity analysis) of the findings.

### Physiological correlates

Although our meta-analysis focused on serum and plasma ADP, some of the papers [32, 34, 36] included urinary ADP (uADP), but the data was too sparse to warrant further analysis. uADP has been linked to renal tubular injury [29]. Thus, uADP may reflect both glomerular and tubular damage in DN. Shimotomai et al. [43] have demonstrated that uADP levels were significantly increased among patients with DN. Such increased levels might result from enhanced filtration of circulating ADP through the damaged kidney. Concordant with our results, Yamamoto et al [44] reported that serum ADP levels are increased in DN patients with MA. However, serum ADP level has been reported to be twice as high in patients with nephrotic syndrome compared to those without, irrespective of glomerular filtration rate [45].

Among type 1 diabetics, patients with MA on average had a higher level of serum ADP when compared to patients with NO and MI, and high ADP levels in urine predicted the progression of MA to ESRD [38]. On the other hand, patients with T1DM, uADP levels were reported to be an independent predictor of DN progression from MA to ESRD. Thus, increase in uADP was identified to be associated with ESRD risk in patients with T1DM [36]. These results indicate that in diabetic patients, an increase in uADP is associated with reduced renal function [46]. Given these physiological correlates, uADP levels maybe a good predictor of DN progression. Diseased states notwithstanding, we need to consider that circulating ADP levels have been positively and independently associated with albuminuria in non-diabetic subjects [47] and that there is a cause–effect relationship between the two variables.

### Strengths and limitations

Interpreting these meta-analysis results warrant awareness of its strengths and limitations. Strengths include: (i) Post-outlier outcomes in zero heterogeneity highlight combinability of the studies; (ii) consistency of significant associations strengthens the evidence for increased ADP associations with DN; (iii) predictive potential of ADP is bolstered by significant AUC under non-heterogeneous conditionsand (iv) all comparisons were deemed robust underpinning the stability of our findings. On the other hand, limitations of our study include: (i) the wide range of patient sample sizes in the studies elicited emphasized contributions from large studies while minimizing those from small studies. This renders caution when interpreting the results; (ii) resulting losses and reductions in heterogeneity from outlier analysis were obtained at the expense of statistical power; where the greater the percentage of omitted studies, the less power is accorded to the resulting post-outlier pooled effects. This is most evident in the outlier-treated subgroups; (iii) issues of multiple comparisons and inadequate statistical power [48] precluded examination of the ethnic subgroups (Asian and Western) as well as ADP isoforms; (iv) ADP may operate in conjunction with other adipocytokines [16, 49] which may likely confound our findings and (v) the relationship established in our study was, for the most part, provided at univariate level of analysis but only from one study at the multivariate level [40].

## Conclusions

In conclusion, our findings indicate that ADP is significantly associated with DN. High significance, non-heterogeneity and stability of the pooled SMDs confirms its associative potential of ADP. In addition, its predictive potential is underpinned by significant AUC values under conditions of non-heterogeneity. Further studies regarding interaction of ADP with other markers and variables may help better understand its role in DN.

## Supporting information

S1 Table Database search algorithms for adiponectin associations with diabetic nephropathy. (DOCX)

S2 Table Assessment of methodological quality of the articles using the Newcastle-Ottawa Scale. (DOCX)

S3 Table Qualitative features of the studies based on methodological, biochemical and clinical characteristics. (DOCX)

S4 Table PRISMA checklist. (DOCX)

S5 Table Evaluating predictive potential of adiponectin in diabetic nephropathy. (DOCX)

S1 List Excluded citations. (DOCX)

## Abbreviations

A: Metric for Area Under the Curve
ACEI: Angiotensin-converting enzyme inhibitors
ACR: Albumin-to-creatinine ratio
ADP: Adiponectin
AER: Albumin excretion rate
AJR: Rodriguez et al. meta-analysis
AM: Analysis model
ARBs: Angiotensin receptor blockers
CC: Case-control
CI: Confidence interval
CN: Control
CS: Cross-sectional
CVD: Cardiovascular disease
df: degree of freedom
DN: Diabetic nephropathy
DT: Diabetes type
EH: Eliminated heterogeneity
ESRD: End-stage renal disease
F: Fixed-effects
GS: Gain in significance
HMS: High to moderate significance
MA: Macroalbuminuria
MHS: Moderate to high significance
MI: Microalbuminuria
n: Number of studies / indicators
NCH: No change in heterogeneity
NM: No mention
NO: Normoalbuminuria
NOS: Newcastle–Ottawa Scale
NSO: Number of studies omitted
P_a_: P-value for association
P_b_: P-value for heterogeneity
PO: Prospective observational
Post: Post-outlier
Pre: Pre-outlier
PRISMA: Preferred Reporting Items for Systematic Reviews and Meta-Analyses
[R]: Reference number
R: Random-effects
RH: Reduced heterogeneity
RHS: Retained high significance
ROC: Receiver Operating Characteristic
SC: Short communication
SD: Standard deviation
SE: Standard error
SMD: Standardized mean difference
T1DM: Type 1 diabetes mellitus
T2DM: Type 2 diabetes mellitus
TZD: Thiazolidinediones
UTI: Urinary tract infection
V: Versus
