# Supplementary material for: Association and biomarker potential of elevated serum adiponectin with nephropathy among type 1 and type 2 diabetics: A meta-analysis
Source: PLoS One. 2018 Dec 17;13(12):e0208905. doi: 10.1371/journal.pone.0208905 (PMC6296550; doi:10.1371/journal.pone.0208905)
Supplement: S1 Table — (DOCX) [file pone.0208905.s001.docx]

**S1 Table. Database search algorithms for adiponectin associations with diabetic nephropathy**

|  | **Database** | **Search strings** | | | |
| --- | --- | --- | --- | --- | --- |
|  | **URL** | **1** | **2** | **3** | **4** |
|  | MEDLINE using PubMed | 190 | 68 | 23 | 38 |
|  | <https://www.ncbi.nlm.nih.gov/pubmed/> |  |  |  |  |
|  | Science Direct | 449 | 388 | 104 | 90 |
|  | <https://www.sciencedirect.com/search> |  |  |  |  |
|  | Google Scholar | 14,600 | 23,300 | 7,450 | 6,120 |
|  | <https://scholar.google.ca/> |  |  |  |  |
|  |  |  |  |  |  |

**String search terms**

1. Adiponectin diabetic nephropathy
2. Serum adiponectin diabetic nephropathy
3. Serum adiponectin diabetic nephropathy albuminuria
4. Urinary adiponectin diabetic nephropathy albuminuria
