# Supplementary material for: Association and biomarker potential of elevated serum adiponectin with nephropathy among type 1 and type 2 diabetics: A meta-analysis
Source: PLoS One. 2018 Dec 17;13(12):e0208905. doi: 10.1371/journal.pone.0208905 (PMC6296550; doi:10.1371/journal.pone.0208905)
Supplement: S2 Table — (DOCX) [file pone.0208905.s002.docx]

**S2** **Table.** **Assessment of methodological quality of the articles using the Newcastle-Ottawa Scale**

|  |  | Selection | | | | Comparability | Exposure | | |  |
| --- | --- | --- | --- | --- | --- | --- | --- | --- | --- | --- |
| Author | Year | Case definition | Representativeness of cases | Selections of controls | Definition of controls | Control for additional factor | Ascertainment of exposure | Same method ascertainment | Non-response rate | **Total Score** |
| Ran | 2010 | 1 | 1 | 1 | 0 | 2 | 1 | 0 | 1 | 7 |
| Fujita | 2006 | 1 | 1 | 0 | 1 | 2 | 0 | 0 | 1 | 6 |
| Kato | 2008 | 1 | 1 | 0 | 1 | 0 | 0 | 0 | 1 | 4 |
| Komaba | 2006 | 1 | 1 | 1 | 0 | 0 | 1 | 0 | 1 | 5 |
| Koshimura | 2004 | 1 | 1 | 1 | 1 | 2 | 0 | 0 | 1 | 7 |
| Saito | 2007 | 1 | 1 | 0 | 1 | 2 | 0 | 0 | 1 | 6 |
| Jorsal | 2013 | 1 | 1 | 1 | 1 | 0 | 0 | 0 | 1 | 5 |
| Schalkwijk | 2006 | 1 | 1 | 1 | 1 | 0 | 0 | 0 | 1 | 5 |
| Panduru | 2015 | 1 | 1 | 1 | 1 | 0 | 1 | 1 | 1 | 7 |
| Saraheimo | 2008 | 1 | 1 | 1 | 1 | 0 | 1 | 0 | 1 | 6 |
| Saraheimo | 2005 | 1 | 1 | 1 | 0 | 2 | 1 | 0 | 1 | 7 |
| Hadjadj | 2005 | 1 | 1 | 1 | 1 | 2 | 1 | 0 | 1 | 8 |
| Yilmaz | 2008 | 1 | 1 | 0 | 1 | 2 | 1 | 1 | 1 | 8 |
|  |  |  |  |  |  |  |  |  |  |  |
