# Supplementary material for: Association and biomarker potential of elevated serum adiponectin with nephropathy among type 1 and type 2 diabetics: A meta-analysis
Source: PLoS One. 2018 Dec 17;13(12):e0208905. doi: 10.1371/journal.pone.0208905 (PMC6296550; doi:10.1371/journal.pone.0208905)
Supplement: S3 Table — (DOCX) [file pone.0208905.s003.docx]

**S3** **Table. Qualitative features of the studies based on methodological, biochemical and clinical characteristics**

| **First author** | **Year** | **Study purpose** | **Addressed ADP isoforms** | **Patient diagnosis** | **Type of study** | **Type of article** | **Patient restrictions** |
| --- | --- | --- | --- | --- | --- | --- | --- |
|  |  |  |  |  |  |  |  |
| Ran | 2010 | Association/Prediction | No | Mogensen’s criteria | CS | Clinical | TZD Glomerular macrovascular UTI |
| Fujita | 2006 | Progression | No | MA | CC | NM | TZD |
| Kato | 2008 | Correlation | Yes | NM | CS | Original | TZD |
| Komaba | 2006 | Association | Yes | ACR | CS | Original | NM |
| Koshimura | 2004 | Association | No | ACR | CC | NM | TZD |
| Saito | 2007 | Association | No | NM | CC | NM | NM |
| Jorsal | 2013 | Association | Yes | AER | CS | NM | NM |
| Schalkwijk | 2006 | Association | Yes | AER | CS | NM | CVD absent, MI MA present |
| Panduru | 2015 | Progression/Prediction | Yes | AER | CC | NM | ESRD |
| Saraheimo | 2008 | Progression/Prediction | No | AER | CC | Original | ESRD |
| Saraheimo | 2005 | Association | No | AER | CC | Original | ESRD |
| Hadjadj | 2005 | Association | No | NM | PO | SC | NM |
| Yilmaz | 2008 | Association/Prediction | No | Proteinuria | CS | Original | Prescribed ACEI, ARBs |
|  |  |  |  |  |  |  |  |

ADP: adiponectin; NM: no mention; MI: microalbuminuria; MA: macroalbuminuria; AER: albumin excretion rate; TZD: thiazolidinediones; UTI: urinary tract infection; CVD: cardiovascular disease; ACR: albumin-to-creatinine ratio; ESRD: end-stage renal disease; ACEI: angiotensin-converting enzyme inhibitors; ARBs: angiotensin receptor blockers; SC: short communication; CS: cross-sectional; CC: case-control; PO: prospective observational
