# Supplementary material for: Association and biomarker potential of elevated serum adiponectin with nephropathy among type 1 and type 2 diabetics: A meta-analysis
Source: PLoS One. 2018 Dec 17;13(12):e0208905. doi: 10.1371/journal.pone.0208905 (PMC6296550; doi:10.1371/journal.pone.0208905)
Supplement: S5 Table — (DOCX) [file pone.0208905.s005.docx]

**S5** **Table. Evaluating predictive potential of adiponectin in diabetic nephropathy**

|  | A | 95% CI | P-value |
| --- | --- | --- | --- |
| CN v MA |  |  |  |
| Pre-outlier | 0.73 | 0.47-0.99 | 0.13 |
| Post- outlier | 0.81 | 0.46-1.16 | 0.15 |
| NO v MI |  |  |  |
| Pre-outlier | 0.62 | 0.37-0.87 | 0.36 |
| Post- outlier | 0.69 | 0.38-1.01 | 0.26 |
| NO v MA |  |  |  |
| Pre-outlier | 0.64 | 0.36-0.91 | 0.32 |
| Post- outlier | **0.88** | **0.68-1.07** | **0.02** |
| MI v MA |  |  |  |
| Pre-outlier | 0.71 | 0.49-0.94 | 0.13 |
| Post- outlier | **0.83** | **0.59-1.10** | **0.05** |
|  |  |  |  |

CN: control; NO: normoalbuminuria; MI: microalbuminuria; MA: macroalbuminuria; v: versus; A: area under the curve; CI: confidence interval
