# Supplementary material for: Association and biomarker potential of elevated serum adiponectin with nephropathy among type 1 and type 2 diabetics: A meta-analysis
Source: PLoS One. 2018 Dec 17;13(12):e0208905. doi: 10.1371/journal.pone.0208905 (PMC6296550; doi:10.1371/journal.pone.0208905)
Supplement: S1 List — (DOCX) [file pone.0208905.s006.docx]

**S1 List. Excluded citations**

**REFERENCES:**

Not human (mice/rat studies) N = 31

1. Cammisotto PG, Londono I, Gingras D, Bendayan M. Control of glycogen synthase through ADIPOR1-AMPK pathway in renal distal tubules of normal and diabetic rats. American journal of physiology Renal physiology. 2008;294(4):F881-9. doi: 10.1152/ajprenal.00373.2007. PubMed PMID: 18256313.

2. Cha DR, Zhang X, Zhang Y, Wu J, Su D, Han JY, et al. Peroxisome proliferator activated receptor alpha/gamma dual agonist tesaglitazar attenuates diabetic nephropathy in db/db mice. Diabetes. 2007;56(8):2036-45. doi: 10.2337/db06-1134. PubMed PMID: 17536062.

3. Choi R, Kim BH, Naowaboot J, Lee MY, Hyun MR, Cho EJ, et al. Effects of ferulic acid on diabetic nephropathy in a rat model of type 2 diabetes. Experimental & molecular medicine. 2011;43(12):676-83. doi: 10.3858/emm.2011.43.12.078. PubMed PMID: 21975281; PubMed Central PMCID: PMC3256295.

4. Fang F, Bae EH, Hu A, Liu GC, Zhou X, Williams V, et al. Deletion of the gene for adiponectin accelerates diabetic nephropathy in the Ins2 (+/C96Y) mouse. Diabetologia. 2015;58(7):1668-78. doi: 10.1007/s00125-015-3605-9. PubMed PMID: 25957229.

5. Gandhi S, Srinivasan B, Akarte AS. Aliskiren improves insulin resistance and ameliorates diabetic renal vascular complications in STZ-induced diabetic rats. Journal of the renin-angiotensin-aldosterone system : JRAAS. 2013;14(1):3-13. doi: 10.1177/1470320312452766. PubMed PMID: 22791702.

6. Gandhi S, Srinivasan BP, Akarte AS. Potential nephrotoxic effects produced by steroidal saponins from hydro alcoholic extract of Tribulus terrestris in STZ-induced diabetic rats. Toxicology mechanisms and methods. 2013;23(7):548-57. doi: 10.3109/15376516.2013.797533. PubMed PMID: 23594260.

7. Guo X, Zhou G, Guo M, Cheung AK, Huang Y, Beddhu S. Adiponectin retards the progression of diabetic nephropathy in db/db mice by counteracting angiotensin II. Physiological reports. 2014;2(2):e00230. doi: 10.1002/phy2.230. PubMed PMID: 24744899; PubMed Central PMCID: PMC3966238.

8. Hou N, Huang N, Han F, Zhao J, Liu X, Sun X. Protective effects of adiponectin on uncoupling of glomerular VEGF-NO axis in early streptozotocin-induced type 2 diabetic rats. International urology and nephrology. 2014;46(10):2045-51. doi: 10.1007/s11255-014-0807-x. PubMed PMID: 25118612.

9. Huang X, Su YX, Deng HC, Zhang MX, Long J, Peng ZG. Suppression of mesangial cell proliferation and extracellular matrix production in streptozotocin-induced diabetic mice by adiponectin in vitro and in vivo. Hormone and metabolic research = Hormon- und Stoffwechselforschung = Hormones et metabolisme. 2014;46(10):736-43. doi: 10.1055/s-0034-1375626. PubMed PMID: 24816832.

10. Jain SK, Croad JL, Velusamy T, Rains JL, Bull R. Chromium dinicocysteinate supplementation can lower blood glucose, CRP, MCP-1, ICAM-1, creatinine, apparently mediated by elevated blood vitamin C and adiponectin and inhibition of NFkappaB, Akt, and Glut-2 in livers of zucker diabetic fatty rats. Molecular nutrition & food research. 2010;54(9):1371-80. doi: 10.1002/mnfr.200900177. PubMed PMID: 20306473; PubMed Central PMCID: PMC3138725.

11. Kang YS, Lee MH, Song HK, Kim JE, Ghee JY, Cha JJ, et al. Chronic Administration of Visfatin Ameliorated Diabetic Nephropathy in Type 2 Diabetic Mice. Kidney & blood pressure research. 2016;41(3):311-24. doi: 10.1159/000443433. PubMed PMID: 27221663.

12. Kang YS, Lee MH, Song HK, Ko GJ, Kwon OS, Lim TK, et al. CCR2 antagonism improves insulin resistance, lipid metabolism, and diabetic nephropathy in type 2 diabetic mice. Kidney international. 2010;78(9):883-94. doi: 10.1038/ki.2010.263. PubMed PMID: 20686445.

13. Kikuchi Y, Yamada M, Imakiire T, Kushiyama T, Higashi K, Hyodo N, et al. A Rho-kinase inhibitor, fasudil, prevents development of diabetes and nephropathy in insulin-resistant diabetic rats. The Journal of endocrinology. 2007;192(3):595-603. doi: 10.1677/JOE-06-0045. PubMed PMID: 17332527.

14. Kobayashi S, Satoh M, Namikoshi T, Haruna Y, Fujimoto S, Arakawa S, et al. Blockade of serotonin 2A receptor improves glomerular endothelial function in rats with streptozotocin-induced diabetic nephropathy. Clinical and experimental nephrology. 2008;12(2):119-25. doi: 10.1007/s10157-007-0011-8. PubMed PMID: 18175064.

15. Kubota M, Watanabe R, Kabasawa H, Iino N, Saito A, Kumagai T, et al. Rice protein ameliorates the progression of diabetic nephropathy in Goto-Kakizaki rats with high-sucrose feeding. The British journal of nutrition. 2013;110(7):1211-9. doi: 10.1017/S0007114513000354. PubMed PMID: 23537514.

16. Lee ES, Lee MY, Kwon MH, Kim HM, Kang JS, Kim YM, et al. Sarpogrelate hydrochloride ameliorates diabetic nephropathy associated with inhibition of macrophage activity and inflammatory reaction in db/db mice. PloS one. 2017;12(6):e0179221. doi: 10.1371/journal.pone.0179221. PubMed PMID: 28640832; PubMed Central PMCID: PMC5480859.

17. Maehira F, Ishimine N, Miyagi I, Eguchi Y, Shimada K, Kawaguchi D, et al. Anti-diabetic effects including diabetic nephropathy of anti-osteoporotic trace minerals on diabetic mice. Nutrition. 2011;27(4):488-95. doi: 10.1016/j.nut.2010.04.007. PubMed PMID: 20708379.

18. Mahfoz AM, El-Latif HA, Ahmed LA, Hassanein NM, Shoka AA. Anti-diabetic and renoprotective effects of aliskiren in streptozotocin-induced diabetic nephropathy in female rats. Naunyn-Schmiedeberg's archives of pharmacology. 2016;389(12):1315-24. doi: 10.1007/s00210-016-1299-2. PubMed PMID: 27612855.

19. Morrison MC, Yakala GK, Liang W, Wielinga PY, Salic K, van Koppen A, et al. Protective effect of rosiglitazone on kidney function in high-fat challenged human-CRP transgenic mice: a possible role for adiponectin and miR-21? Scientific reports. 2017;7(1):2915. doi: 10.1038/s41598-017-02444-2. PubMed PMID: 28588299; PubMed Central PMCID: PMC5460286.

20. Nakamaki S, Satoh H, Kudoh A, Hayashi Y, Hirai H, Watanabe T. Adiponectin reduces proteinuria in streptozotocin-induced diabetic Wistar rats. Experimental biology and medicine. 2011;236(5):614-20. doi: 10.1258/ebm.2011.010218. PubMed PMID: 21521713.

21. Nam DH, Lee MH, Kim JE, Song HK, Kang YS, Lee JE, et al. Blockade of cannabinoid receptor 1 improves insulin resistance, lipid metabolism, and diabetic nephropathy in db/db mice. Endocrinology. 2012;153(3):1387-96. doi: 10.1210/en.2011-1423. PubMed PMID: 22234468.

22. Ndisang JF, Jadhav A. Hemin therapy improves kidney function in male streptozotocin-induced diabetic rats: role of the heme oxygenase/atrial natriuretic peptide/adiponectin axis. Endocrinology. 2014;155(1):215-29. doi: 10.1210/en.2013-1050. PubMed PMID: 24140713.

23. Ndisang JF, Jadhav A, Mishra M. The heme oxygenase system suppresses perirenal visceral adiposity, abates renal inflammation and ameliorates diabetic nephropathy in Zucker diabetic fatty rats. PloS one. 2014;9(1):e87936. doi: 10.1371/journal.pone.0087936. PubMed PMID: 24498225; PubMed Central PMCID: PMC3907578.

24. Noda K, Melhorn MI, Zandi S, Frimmel S, Tayyari F, Hisatomi T, et al. An animal model of spontaneous metabolic syndrome: Nile grass rat. FASEB journal : official publication of the Federation of American Societies for Experimental Biology. 2010;24(7):2443-53. doi: 10.1096/fj.09-152678. PubMed PMID: 20335226; PubMed Central PMCID: PMC2887270.

25. Ohtomo S, Izuhara Y, Nangaku M, Dan T, Ito S, van Ypersele de Strihou C, et al. Body weight control by a high-carbohydrate/low-fat diet slows the progression of diabetic kidney damage in an obese, hypertensive, type 2 diabetic rat model. Journal of obesity. 2010;2010. doi: 10.1155/2010/136502. PubMed PMID: 20700413; PubMed Central PMCID: PMC2911582.

26. Ojima A, Matsui T, Nakamura N, Higashimoto Y, Ueda S, Fukami K, et al. DNA aptamer raised against advanced glycation end products (AGEs) improves glycemic control and decreases adipocyte size in fructose-fed rats by suppressing AGE-RAGE axis. Hormone and metabolic research = Hormon- und Stoffwechselforschung = Hormones et metabolisme. 2015;47(4):253-8. doi: 10.1055/s-0034-1385904. PubMed PMID: 25105541.

27. Proctor SD, Kelly SE, Stanhope KL, Havel PJ, Russell JC. Synergistic effects of conjugated linoleic acid and chromium picolinate improve vascular function and renal pathophysiology in the insulin-resistant JCR:LA-cp rat. Diabetes, obesity & metabolism. 2007;9(1):87-95. doi: 10.1111/j.1463-1326.2006.00578.x. PubMed PMID: 17199723.

28. Ragolia L, Palaia T, Hall CE, Maesaka JK, Eguchi N, Urade Y. Accelerated glucose intolerance, nephropathy, and atherosclerosis in prostaglandin D2 synthase knock-out mice. The Journal of biological chemistry. 2005;280(33):29946-55. doi: 10.1074/jbc.M502927200. PubMed PMID: 15970590.

29. Tamura Y, Murayama T, Minami M, Matsubara T, Yokode M, Arai H. Ezetimibe ameliorates early diabetic nephropathy in db/db mice. Journal of atherosclerosis and thrombosis. 2012;19(7):608-18. PubMed PMID: 22498767.

30. Williams KJ, Qiu G, Usui HK, Dunn SR, McCue P, Bottinger E, et al. Decorin deficiency enhances progressive nephropathy in diabetic mice. The American journal of pathology. 2007;171(5):1441-50. doi: 10.2353/ajpath.2007.070079. PubMed PMID: 17884968; PubMed Central PMCID: PMC2043506.

31. Yuan F, Liu YH, Liu FY, Peng YM, Tian JW. Intraperitoneal administration of the globular adiponectin gene ameliorates diabetic nephropathy in Wistar rats. Molecular medicine reports. 2014;9(6):2293-300. doi: 10.3892/mmr.2014.2133. PubMed PMID: 24718692.

Reviews/meta-analyses N = 11

32. Bayliss G, Weinrauch LA, D'Elia JA. Pathophysiology of obesity-related renal dysfunction contributes to diabetic nephropathy. Current diabetes reports. 2012;12(4):440-6. doi: 10.1007/s11892-012-0288-1. PubMed PMID: 22638939.

33. Bell DS, DiNicolantonio JJ, O'Keefe JH. Is statin-induced diabetes clinically relevant? A comprehensive review of the literature. Diabetes, obesity & metabolism. 2014;16(8):689-94. doi: 10.1111/dom.12254. PubMed PMID: 24373206.

34. Christou GA, Kiortsis DN. The role of adiponectin in renal physiology and development of albuminuria. The Journal of endocrinology. 2014;221(2):R49-61. doi: 10.1530/JOE-13-0578. PubMed PMID: 24464020.

35. Freedman BI, Bostrom M, Daeihagh P, Bowden DW. Genetic factors in diabetic nephropathy. Clinical journal of the American Society of Nephrology : CJASN. 2007;2(6):1306-16. doi: 10.2215/CJN.02560607. PubMed PMID: 17942768.

36. Granier C, Makni K, Molina L, Jardin-Watelet B, Ayadi H, Jarraya F. Gene and protein markers of diabetic nephropathy. Nephrology, dialysis, transplantation : official publication of the European Dialysis and Transplant Association - European Renal Association. 2008;23(3):792-9. doi: 10.1093/ndt/gfm834. PubMed PMID: 18065784.

37. Jim B, Santos J, Spath F, Cijiang He J. Biomarkers of diabetic nephropathy, the present and the future. Current diabetes reviews. 2012;8(5):317-28. PubMed PMID: 22698077.

38. Khodaeian M, Enayati S, Tabatabaei-Malazy O, Amoli MM. Association between Genetic Variants and Diabetes Mellitus in Iranian Populations: A Systematic Review of Observational Studies. Journal of diabetes research. 2015;2015:585917. doi: 10.1155/2015/585917. PubMed PMID: 26587547; PubMed Central PMCID: PMC4637497.

39. Rodriguez AJ, Nunes Vdos S, Mastronardi CA, Neeman T, Paz-Filho GJ. Association between circulating adipocytokine concentrations and microvascular complications in patients with type 2 diabetes mellitus: A systematic review and meta-analysis of controlled cross-sectional studies. Journal of diabetes and its complications. 2016;30(2):357-67. doi: 10.1016/j.jdiacomp.2015.11.004. PubMed PMID: 26684169.

40. Yang J, Zhang D, Li J, Zhang X, Fan F, Guan Y. Role of PPARgamma in renoprotection in Type 2 diabetes: molecular mechanisms and therapeutic potential. Clinical science. 2009;116(1):17-26. doi: 10.1042/CS20070462. PubMed PMID: 19037881.

41. Zha D, Wu X, Gao P. Adiponectin and Its Receptors in Diabetic Kidney Disease: Molecular Mechanisms and Clinical Potential. Endocrinology. 2017;158(7):2022-34. doi: 10.1210/en.2016-1765. PubMed PMID: 28402446.

42. Zoccali C, Mallamaci F. Obesity, diabetes, adiponectin and the kidney: a podocyte affair. Nephrology, dialysis, transplantation : official publication of the European Dialysis and Transplant Association - European Renal Association. 2008;23(12):3767-70. doi: 10.1093/ndt/gfn517. PubMed PMID: 18802210.

Genetic studies N = 7

43. Bostrom MA, Freedman BI, Langefeld CD, Liu L, Hicks PJ, Bowden DW. Association of adiponectin gene polymorphisms with type 2 diabetes in an African American population enriched for nephropathy. Diabetes. 2009;58(2):499-504. doi: 10.2337/db08-0598. PubMed PMID: 19056609; PubMed Central PMCID: PMC2628626.

44. Choe EY, Wang HJ, Kwon O, Kim KJ, Kim BS, Lee BW, et al. Variants of the adiponectin gene and diabetic microvascular complications in patients with type 2 diabetes. Metabolism: clinical and experimental. 2013;62(5):677-85. doi: 10.1016/j.metabol.2012.11.005. PubMed PMID: 23260797.

45. Chung HF, Long KZ, Hsu CC, Mamun AA, Chiu YF, Tu HP, et al. Adiponectin gene (ADIPOQ) polymorphisms correlate with the progression of nephropathy in Taiwanese male patients with type 2 diabetes. Diabetes research and clinical practice. 2014;105(2):261-70. doi: 10.1016/j.diabres.2014.04.015. PubMed PMID: 24894086.

46. El-Shal AS, Zidan HE, Rashad NM. Adiponectin gene polymorphisms in Egyptian type 2 diabetes mellitus patients with and without diabetic nephropathy. Molecular biology reports. 2014;41(4):2287-98. doi: 10.1007/s11033-014-3082-0. PubMed PMID: 24469713.

47. Ma J, Mollsten A, Falhammar H, Brismar K, Dahlquist G, Efendic S, et al. Genetic association analysis of the adiponectin polymorphisms in type 1 diabetes with and without diabetic nephropathy. Journal of diabetes and its complications. 2007;21(1):28-33. doi: 10.1016/j.jdiacomp.2006.03.002. PubMed PMID: 17189871.

48. Sikka R, Raina P, Matharoo K, Bandesh K, Bhatia R, Chakrabarti S, et al. TNF-alpha (g.-308 G > A) and ADIPOQ (g. + 45 T > G) gene polymorphisms in type 2 diabetes and microvascular complications in the region of Punjab (North-West India). Current eye research. 2014;39(10):1042-51. doi: 10.3109/02713683.2014.892998. PubMed PMID: 24655058.

49. Wu LS, Hsieh CH, Pei D, Hung YJ, Kuo SW, Lin E. Association and interaction analyses of genetic variants in ADIPOQ, ENPP1, GHSR, PPARgamma and TCF7L2 genes for diabetic nephropathy in a Taiwanese population with type 2 diabetes. Nephrology, dialysis, transplantation : official publication of the European Dialysis and Transplant Association - European Renal Association. 2009;24(11):3360-6. doi: 10.1093/ndt/gfp271. PubMed PMID: 19506043.

Not diabetic nephropathy not adiponectin N = 8

50. Ahmad J, Zubair M, Malik A, Siddiqui MA, Wangnoo SK. Cathepsin-D, adiponectin, TNF-alpha, IL-6 and hsCRP plasma levels in subjects with diabetic foot and possible correlation with clinical variables: a multicentric study. Foot. 2012;22(3):194-9. doi: 10.1016/j.foot.2012.03.015. PubMed PMID: 22560191.

51. Dossarps D, Petit JM, Guiu B, Cercueil JP, Duvillard L, Bron AM, et al. Body fat distribution and adipokine secretion are not associated with diabetic retinopathy in patients with type 2 diabetes mellitus. Ophthalmic research. 2014;51(1):42-5. doi: 10.1159/000355323. PubMed PMID: 24217637.

52. Jung CH, Kim BY, Kim CH, Kang SK, Jung SH, Mok JO. Associations of serum fetuin-A levels with insulin resistance and vascular complications in patients with type 2 diabetes. Diabetes & vascular disease research. 2013;10(5):459-67. doi: 10.1177/1479164113490766. PubMed PMID: 23811603.

53. Jung CH, Kim BY, Mok JO, Kang SK, Kim CH. Association between serum adipocytokine levels and microangiopathies in patients with type 2 diabetes mellitus. Journal of diabetes investigation. 2014;5(3):333-9. doi: 10.1111/jdi.12144. PubMed PMID: 24843783; PubMed Central PMCID: PMC4020339.

54. Kampe K, Sieber J, Orellana JM, Mundel P, Jehle AW. Susceptibility of podocytes to palmitic acid is regulated by fatty acid oxidation and inversely depends on acetyl-CoA carboxylases 1 and 2. American journal of physiology Renal physiology. 2014;306(4):F401-9. doi: 10.1152/ajprenal.00454.2013. PubMed PMID: 24338821; PubMed Central PMCID: PMC3920022.

55. Kopf S, Oikonomou D, von Eynatten M, Kieser M, Zdunek D, Hess G, et al. Urinary excretion of high molecular weight adiponectin is an independent predictor of decline of renal function in type 2 diabetes. Acta diabetologica. 2014;51(3):479-89. doi: 10.1007/s00592-013-0542-2. PubMed PMID: 24366425.

56. Mao D, Peng H, Li Q, Wang J, Li P, Hu K, et al. Aqueous humor and plasma adiponectin levels in proliferative diabetic retinopathy patients. Current eye research. 2012;37(9):803-8. doi: 10.3109/02713683.2012.676700. PubMed PMID: 22563689.

57. Tesauro M, Canale MP, Rodia G, Di Daniele N, Lauro D, Scuteri A, et al. Metabolic syndrome, chronic kidney, and cardiovascular diseases: role of adipokines. Cardiology research and practice. 2011;2011:653182. doi: 10.4061/2011/653182. PubMed PMID: 21403882; PubMed Central PMCID: PMC3051177.

**Screened citations from full-text (5)**

**No suitable data**

58. Aso Y, Suganuma R, Wakabayashi S, Hara K, Nakano T, Suetsugu M, et al. Anemia is associated with an elevated serum level of high-molecular-weight adiponectin in patients with type 2 diabetes independently of renal dysfunction. Translational research : the journal of laboratory and clinical medicine. 2009;154(4):175-82. doi: 10.1016/j.trsl.2009.07.005. PubMed PMID: 19766961.

59. Bjornstad P, Pyle L, Kinney GL, Rewers M, Johnson RJ, Maahs DM, et al. Adiponectin is associated with early diabetic kidney disease in adults with type 1 diabetes: A Coronary Artery Calcification in Type 1 Diabetes (CACTI) Study. Journal of diabetes and its complications. 2017;31(2):369-74. doi: 10.1016/j.jdiacomp.2016.06.012. PubMed PMID: 27368123; PubMed Central PMCID: PMC5156602.

60. Choi SR, Lim JH, Kim MY, Kim EN, Kim Y, Choi BS, et al. Adiponectin receptor agonist AdipoRon decreased ceramide, and lipotoxicity, and ameliorated diabetic nephropathy. Metabolism: clinical and experimental. 2018;85:348-60. doi: 10.1016/j.metabol.2018.02.004. PubMed PMID: 29462574.

61. Guo LL, Pan Y, Jin HM. Adiponectin is positively associated with insulin resistance in subjects with type 2 diabetic nephropathy and effects of angiotensin II type 1 receptor blocker losartan. Nephrology, dialysis, transplantation : official publication of the European Dialysis and Transplant Association - European Renal Association. 2009;24(6):1876-83. doi: 10.1093/ndt/gfn770. PubMed PMID: 19164322.

62. Ohashi N, Kato A, Misaki T, Sakakima M, Fujigaki Y, Yamamoto T, et al. Association of serum adiponectin levels with all-cause mortality in hemodialysis patients. Internal medicine. 2008;47(6):485-91. PubMed PMID: 18344634.
